# A Review on Humidity, Temperature and Strain Printed Sensors—Current Trends and Future Perspectives

**DOI:** 10.3390/s21030739

**Published:** 2021-01-22

**Authors:** Dimitris Barmpakos, Grigoris Kaltsas

**Affiliations:** 1 microSENSES Laboratory, Department of Electrical and Electronics Engineering, University of West Attica, Ancient Olive-Grove Campus, 12243 Athens, Greece; g.kaltsas@uniwa.gr; 2 Institute of Nanoscience and Nanotechnology, National Centre for Scientific Research “Demokritos”, P.O. Box 60037, Agia Paraskevi, 15310 Athens, Greece; 3 Physics Department, University of Patras, Rion, 26504 Patras, Greece

**Keywords:** printed humidity sensor, printed temperature sensor, printed strain sensor, flexible sensor, multi-material sensor, printed electronics, flexible electronics

## Abstract

Printing technologies have been attracting increasing interest in the manufacture of electronic devices and sensors. They offer a unique set of advantages such as additive material deposition and low to no material waste, digitally-controlled design and printing, elimination of multiple steps for device manufacturing, wide material compatibility and large scale production to name but a few. Some of the most popular and interesting sensors are relative humidity, temperature and strain sensors. In that regard, this review analyzes the utilization and involvement of printing technologies for full or partial sensor manufacturing; production methods, material selection, sensing mechanisms and performance comparison are presented for each category, while grouping of sensor sub-categories is performed in all applicable cases. A key aim of this review is to provide a reference for sensor designers regarding all the aforementioned parameters, by highlighting strengths and weaknesses for different approaches in printed humidity, temperature and strain sensor manufacturing with printing technologies.

## 1. Introduction

**Figure 1:**
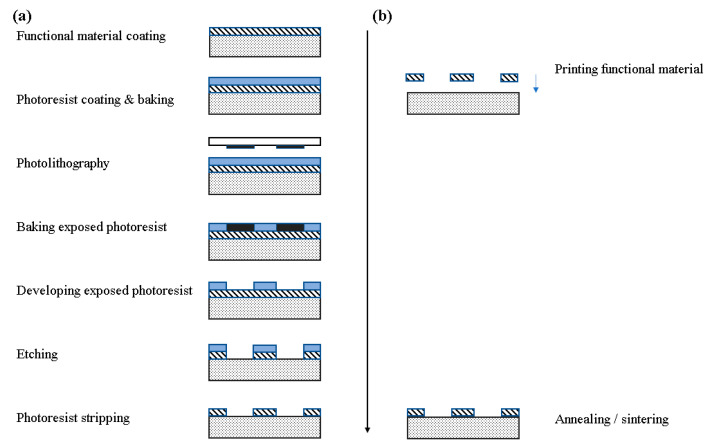
Comparison of Si-based manufacturing and printing process. (**a**) Traditional IC manufacturing; (**b**) printing process.

Some of the most interesting physical sensors which find a wide range of applications are relative humidity [1], temperature [2] and strain [3] sensors; they can be used in indoor or outdoor applications, in on-body measurements and other biomedical settings, in environmental, agricultural, room monitoring and so on. For developing these sensors to meet modern application demands, requirements for mass scale, cost-effective approaches with smart material combinations arise. Manufacturing techniques such as printing technologies for sensor development are a blooming field which dynamically responds to these demands. Printed electronics are a type of electronic devices, manufactured by a usually additive printing process, i.e., direct material deposition onto a substrate in a patterned manner, or more formally an electronic science and technology based on conventional printing techniques as the means to manufacture electronic devices and systems [4]. A generalized comparison between traditional IC and additive manufacturing [4] is presented in Figure 1. It is obvious that the required steps to manufacture a device are much less complex and straightforward when utilizing an additive process. Also, no etching is required, providing two more advantages: (a) no active material loss and (b) little to no byproducts and resulting chemical waste.

Summarizing the benefits of sensor development with printing technologies, the key advantages are reduced production cost (approximately 1/10–1/100 of an investment compared to that of Si technology), reduced production time, increased manufacturing area, flexibility—mechanical endurance and multiple material—substrate combinations with one piece of equipment [5].

The Organic and Printed Electronics Association (OE-A) has recently released a development roadmap [6] which includes the main sectors that printed electronics are expected to dominate in the following years:

- IoT: Optimized maintenance of buildings, smart labels for logistics, environmental monitoring, sensors for material monitoring, energy management, autonomous sensors, heating elements.
- Healthcare: Medical packages, patches for therapy and monitoring, biomarker diagnosis, OLEDs, smart wound treatment
- Automotive: OLED lighting, flexible and OLED displays for mirrors and HMI (Human-Machine Interface), sensors.
- Consumer electronics: Flexible displays and sensors, curved touch surfaces with sensors, smart wearables, memories, batteries, RFID tags.

All these categories include sensor applications of the three aforementioned physical quantities in different fields. It is therefore mandatory to investigate the current status of such important developments in the sensing research community.

**Figure 2:**
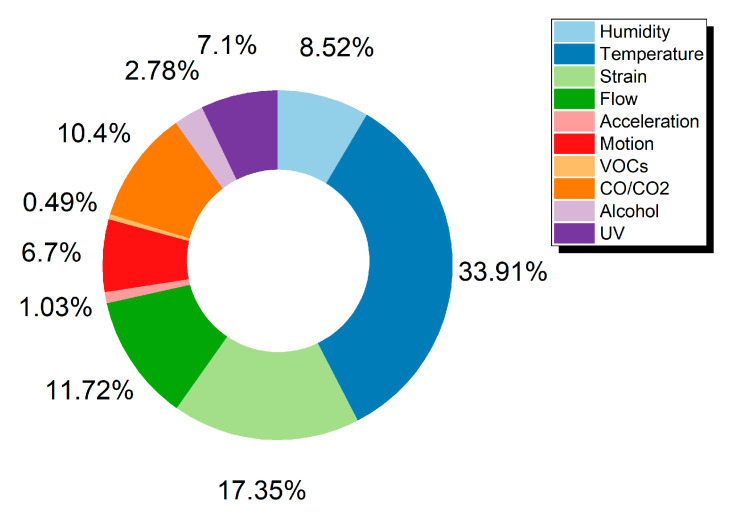
Publications for printed sensors categories between 2013–2020.

**Figure 3:**
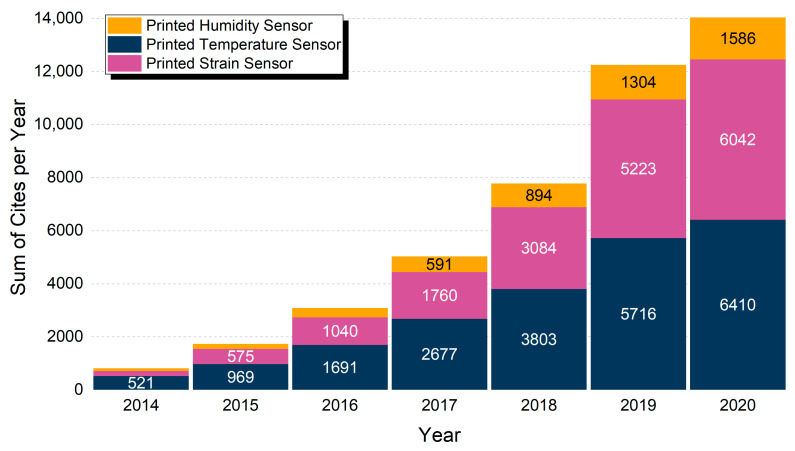
Total citations per year in the range 2014–2020 for “Printed Humidity Sensor”, “Printed Temperature Sensor” and “Printed Strain Sensor”. Year 2013 had less than 200 items and is not displayed.

By gathering data from the Web of Science Core Collection^TM^ (Clarivate^TM^, Philadelphia, PA, USA) an overview of the current trends for various sensors was acquired and is presented in Figure 2 and Figure 3 and Table 1. The Web of Science database is considered to include publications of high scientific impact and solely scientific publications in contrast to other databases [7]. Out of a total of 4462 publications, the percentage of each sensor category is extracted and presented in Figure 2, amongst the most commonly presented sensors in the literature for the years 2013 to 2020. For the scope of this review, the authors present three categories which show a steady increase in attracting more research and present a variety of approaches in manufacturing via printing techniques and materials utilization. IDTechEx forecasts the market for fully printed sensors will be over $8 billion by 2025 [8]. According to the same report the printed sensors field will grow fast and the corresponding Compound Annual Growth Rate (CAGR) for the 2015–2025 period, is predicted to follow a trend, where humidity and temperature sensors demonstrate the highest growth rate.

The search was conducted on December 2020, while queries served by the database are presented in Figure 2 legend, accompanied with the terms “printed sensor” (e.g., “printed humidity sensor”, “printed temperature sensor” and so on). The data extracted is for the year range 2013–2020.

It is obvious that there exists an increasing trend in all three fields, with temperature and strain sensors sharing approximately equal number of citations, but with strain sensors having approximately half the publications (Figure 3). This indicates amongst others, (a) the heavy scientific importance of development of such sensors and the overall high research interest that these devices pose and (b) a set of applications that correlates these devices, but with printed temperature sensors reaching a more mature state. Printed humidity sensors have a lower number of publications and citations; nevertheless, the growth rate is similar, indicating that interest in these sensors is ever-increasing, and can also indicate a different state of innovation, whether it is material novelty, patterning methods and geometries and so on.

**Figure 4:**
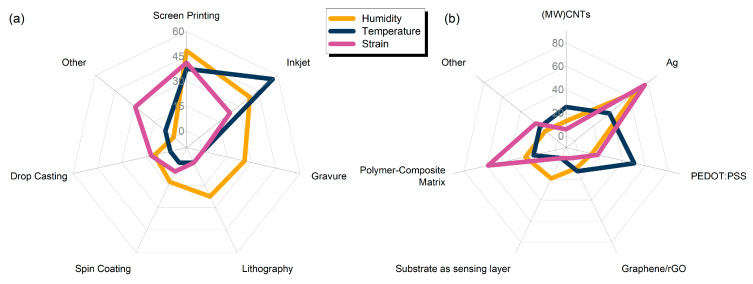
(**a**) Radar plot with utilization percentage values for different fabrication techniques for all the sensor categories; (**b)** material selection for electrodes and sensing layers.

A key common denominator for all the presented works herein is the direct involvement of a printing process for either full or partial development of sensors. Some statistics regarding the distribution of printing technique are presented in Figure 4a, from 31 publications regarding humidity, 16 regarding temperature and 17 regarding strain sensors. The dominant technologies for either film or electrode patterning are screen printing (48%, 37% and 41% for humidity, temperature and strain sensor development) followed by inkjet printing (38%, 56% and 23% for humidity, temperature and strain sensor development). It should be noted here that this graph solely serves as an indicator for involvement of a printing process in the overall fabrication development; that is, a variety of published works analyzed herein utilize multiple techniques for sensor development. It is therefore crucial to include non-printing techniques such as traditional lithography in the overview.

Material selection is vital in sensor design; as presented in Figure 4b, silver is used in the vast majority of designs for all three sensor categories, mostly for electrode patterning. Humidity sensing can be performed by using substrate as sensing layer, as it will be presented in the humidity sensors analysis. Strain sensors largely rely on polymers and polymer composites, for their inherent flexibility. Different research groups incorporate PEDOT: PSS for temperature sensing; this conductive polymer presents a set of advantages, namely, low cost, biocompatibility, optical transparency and plasticity. 

This review presents and assesses the recent developments in printed humidity, temperature and strain sensors design, material selection, fabrication, characterization and evaluation. For each sensor family, the appropriate categories have been identified and a corresponding analysis has been performed. Material selection approaches, performance, fabrication strategies and design considerations have been thoroughly discussed resulting in a comparison with the intend to pave a guideline for assisting in selection of all the aforementioned parameters and furthermore to demonstrate the state of the art in each category.

## 2. Printed Humidity Sensors

Humidity sensors realized with printing technologies offer drastically lower costs and increased capabilities for large scale production. A general approach for low-complexity devices is based on the deposition of a single material on a substrate and exploitation of these two materials’ physical properties for sensing (Figure 4, substrate as sensing film). On the other hand, in traditionally developed sensors, conductive electrodes are utilized on a substrate for measuring response of an active film, usually deposited or developed onto the electrodes (Figure 4, other coating methods for sensing film). Principle of operation is similar for both cases; water molecules are absorbed from either the substrate (in the first case) or the active film (in the second case); in both cases they diffuse in the corresponding layer, therefore inducing changes in its electrical properties. So, we can note that in the first case the substrate itself acts as a sensing layer, eliminating the need for an additional sensing layer. This approach usually has a negative impact on performance; therefore, the sensor designer should always consider the tradeoffs before proceeding to implementation. Regarding sensing mechanisms discussed herein, it should be mentioned that humidity (broadly described) influences the conducting mechanism of a given material. This chapter presents various materials whose electrical properties alternate with humidity and examples will be given per case. Nevertheless, the vast majority of designs incorporates a set of interdigitated electrodes (IDEs), with various finger lengths (L), finger spacings (d) and finger widths (W).

The term sensor memory effect (lag, or hysteresis) is used to describe a common problem in sensors mainly due to absorption, such as the humidity sensors discussed here. During exposure to humidity, the sensor absorbs water molecules, which induces a change of an electrical parameter, leading to successful detection of humidity levels; the problem arises when the sensor is required to desorb the previously absorbed water molecules. Various materials are designed for high water absorption such as TiO_2_ nanoparticle films [9] and MWCNTs [10] that offer a high surface area to volume ratio, which in turn leads to difficult desorption of trapped water molecules without external assistance. There are two approaches in tackling this problem: one being the careful planning and material selection, for finding material combinations that favor fast and repeatable absorption-desorption cycles, and the other incorporating an active element serving as heater [10], which assists in humidity desorption. Returning to our initial consideration for low-complexity, low-cost and mass production of a sensor, the incorporation of a heater does not converge with the original requirements, given the fact that it also requires active electronics for driving the heater. 

**Figure 5:**
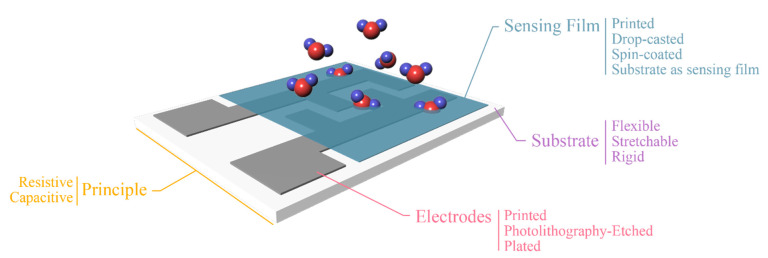
Various setups of partially of fully printed humidity sensors. Each category represents a subset of design considerations.

Another consideration is the sensor readout, which can be either resistive or capacitive, meaning that variations in environmental relative humidity can be directly reflected to the terminal pads (as denoted in Figure 5) during the measurement of the corresponding electrical signal. Measuring electrical resistance implements fewer and simpler electronics, while for an accurate capacitance measurement more sophisticated electronics are required. Alternatively, the sensor can be incorporated in an antenna array, and its detuning can be matched with relative humidity, in a powerless RFID manner [11,12]. 

Material selection is of vital importance in sensor performance; conductive electrodes can be printed with Ag [11,13,14,15,16] or PEDOT:PSS [17] and flexible substrates utilized can be PI (Kapton) [10,14,15,18,19], PET [16,20,21,22], polyester-based [23,24], paper [12,17]; or rigid, such as glass [25] or ceramic [26]; this is directly correlated to the printing technique. A hybrid approach has been proposed by numerous research groups, where the conductive electrodes are not printed but traditionally patterned and the sensing layer is printed on top [19,26,27,28]. In this section we will group sensors in categories based on the substrate utilized, because in the bottom line it is that parameter which indicates the application range: paper cannot be heated above a certain temperature, prohibiting annealing of an active layer, while polyimide, provided by DuPont under its commercial line Kapton, is extensively utilized for development of flexible electronics and sensors, due to its high durability in harsh environments and its high glass transition temperature (as high as 400 °C [29]), allowing for a wide variety of printed materials. PET is widely used for its transparency, making it suitable for a variety of applications. It should be noted that some sensors presented herein utilize drop-cast or spin coating for material deposition. These deposition techniques can be directly replaced by a printing process; therefore the specific sensors are included in the review for highlighting the material selection of each work, all towards a fully additive manufacturing approach. In order to create comparison graphs from the data extracted of each source, WebPlotDigitizer has been used [30].

### 2.1. Resistive Printed Humidity Sensors

This section analyzes recent advances in resistive type humidity sensors fabricated with printing techniques; resistive output sensors provide a straightforward interface with measurement electronics. As mentioned above, they can either incorporate a set of electrodes and a sensing film deposited on top, or exploit the substrate’s electrical response to humidity variations for humidity detection. The analyzed papers are categorized based on the substrate utilized, e.g., Kapton, PET, and other substrates such as LiNbO_3_ and paper.

#### 2.1.1. Kapton—Based Resistive Humidity Sensors 

**Figure 6:**
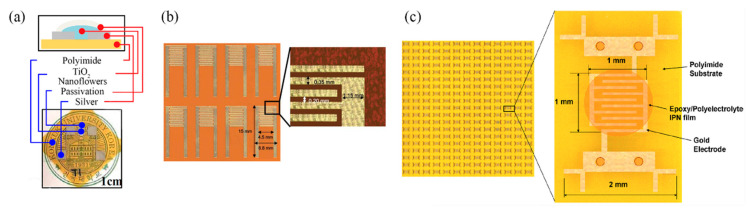
(**a**) A gravure-printed set of Ag electrodes with a drop-cased TiO_2_ nanoflower sensing film (reproduced with permission from [14] copyright 2019, Elsevier); (**b**) screen printing and electroless plating of electrodes on Kapton (reproduced with permission from [15] copyright 2012, The Royal Society of Chemistry); (**c**) screen-printable active film deposition of Au electrodes, on Kapton substrate (reproduced with permission from [18] copyright 2013, Elsevier).

Zhang et al. [10] developed a MWCNT film with gravure printing on Kapton, with overlayed Ag screen printed electrodes, incorporating a back-plane Ag screen printed heater as well, for tackling the aforementioned hysteresis problem in the range of 30–60% RH with a resistivity of 0.96%/% RH (12 to 17 kΩ increase for full working range); evolution of this research resulted in a wider range (10–90% RH) with a total resistance change of 40.0 ± 1.7% from 20 to 80% RH [13]. Jeong et al. [14] gravure printed Ag electrodes and drop-casted TiO2 nanoflowers as a sensing layer for achieving a sensitivity of 485.7 RH%^−1^ between 20 and 95% RH (Figure 6a). Kim and Gong [15] fabricated gold electrodes by screen printing silver and electroless plating, followed by screen printing a novel photocured copolymer (MEPAB/CMDAB/MMA) for humidity sensing, offering great reliability with resistance to water, showing minimal change in behavior when soaked in water for up to 60 min (Figure 6b). 

Lim et al. [18] developed a screen-printable humidity sensing film consisting of two parts: a thermally curable epoxy resin and a photocurable polyelectrolyte. This combination (interpenetrating polymer network—IPN) is investigated for its adhesion to polyimide substrate, because sole polyelectrolyte humidity-sensitive films present poor anchoring to polyimide (Figure 6c). The following mechanism was exploited for humidity sensing in [19]: SnO_2_/rGO drop-casted films resistance presented an increase with relative humidity; it is known that rGO is a p-type nanomaterial, while adsorbed water molecules act as electron donors, therefore there are two mechanisms acting concurrently for resistance increase: decrease of hole concentration due to water molecules and swelling of rGO which in turn leads to higher interlayer resistance between SnO_2_ and rGO.

#### 2.1.2. PET—Based Resistive Humidity Sensors

Ali et al. [20] proposed a graphene/methyl—red sensing layer over inkjet-printed Ag electrodes on PET substrate; the mechanism for humidity detection is described to depend on the graphene flake-to-flake electrical connection via methyl-red; water vapor absorption from methyl-red leads to better electrical connection, therefore the overall sensor resistance falls with higher humidity.

**Figure 7:**
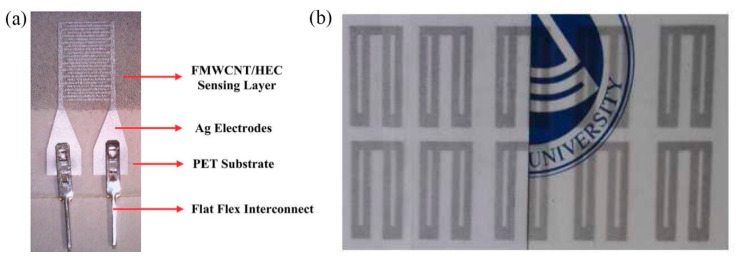
(**a**) FMWCNT/HEC on Ag electrodes (reproduced with permission from [21] copyright 2019, The Royal Society of Chemistry); (**b**) high-transparent PEDOT/rGO/PEI-Au NP printed geometry with good mechanical endurance (reproduced with permission from [22] copyright 2018, Elsevier).

A humidity-sensitive carbon nanotube (CNT) film was deposited via gravure printing on a set of screen-printed Ag electrodes [16] for successfully measuring relative humidity in the range 20 to 80% RH. Gravure printing was also utilized for depositing a functionalized multi-walled carbon nanotube (FMWCNTs)/hydroxyethyl cellulose (HEC) composite onto screen printed electrodes [21] (Figure 7a). This composite relies on two mechanisms for enhanced sensor response: on the one hand FMWCNTs, demonstrating p-type semiconductor characteristics, exhibit an increase in resistance driven by transfer of more electrons, due to adsorption via physisorption. Additionally, FMWCNTs owning defective hydroxyl and carboxyl groups exhibit large hydrophilicity, enhancing interaction with water molecules, and therefore aiding to electron transfer (resistance increase). In parallel, HEC swells when exposed to humidity; therefore, the contact gap of FMWCNTs increases, further contributing to resistance increase. Zhang et al. [22] recently proposed a poly(3,4-ethylenedioxythiophene) (PEDOT)/reduced graphene oxide (rGO)/ Au NP modified with polyethyleneimine (PEI) ink for humidity sensing; high transparency, electrical performance and sensitivity were observed even after 200 bending cycles, while the sensor was capable of detecting variations in humidity in the range of 11–98% RH (Figure 7b).

#### 2.1.3. Resistive Humidity Sensors on Other Substrates

**Figure 8:**
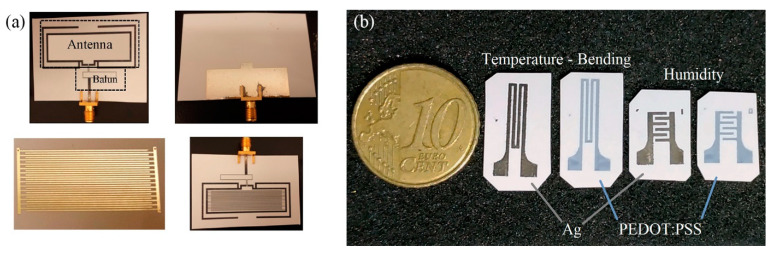
(**a**) Paper-based resistive humidity sensors: exploitation of substrate resistivity variations via antenna detuning for a passive RFID sensor node (reproduced with permission from [12] copyright 2016, MDPI AG); (**b**) PEDOT:PSS and Ag NP paper-based resistive humidity device on paper (reproduced with permission from [17] copyright 2020, Elsevier).

Surface acoustic waves (SAW) have been proven to assist both absorption and desorption of sensors developed on piezoelectric substrate; more specifically, 1 kHz waves used with sensors developed on LiNbO_3_ substrate with different PEDOT: PSS-based sensing materials yielded good response for a wide range of relative humidity (0–90% RH) [27,28]. Paper-based resistive humidity sensors have also been recently presented; these devices utilize cellulose electrical properties modification by high water molecule absorption [12,17]; this approach provides a very cost-effective design guideline, where mass production of low-cost devices is enabled by simply patterning commercial paper with a conductive ink (Figure 8a,b). Additionally, a paper-based sensor consisting of inkjet-printed graphene oxide sensing on screen-printed graphene electrodes was demonstrated, with a response time of 6.5 and 2.4 s, respectively [31].

#### 2.1.4. Resistive Humidity Sensors Comparison

For a comprehensive performance overview of the devices, the most crucial fabrication parameters alongside some performance characteristics are presented in Table 2.

**Figure 9:**
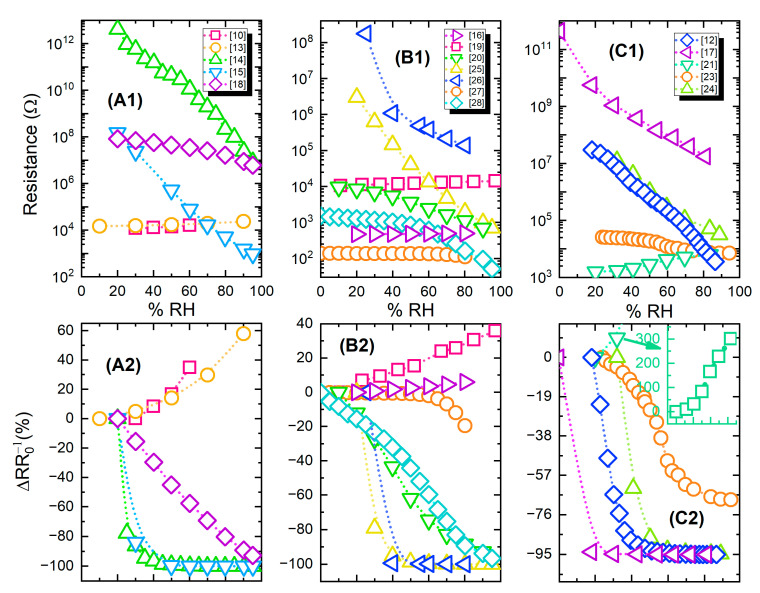
(**A1**–**C1**); Resistive humidity sensors response (resistance value) for different relative humidity levels; (**A2**–**C2**) relative percentage change for the same sensors. The graphs share the same legends and X axes per vertical couple, i.e., A1 & A2 have the same legend.

Figure 9 presents both absolute and relative resistance change (% response) for groups of devices from Table 2; the information has been divided into three parts for representation clarity.

### 2.2. Capacitive Printed Humidity Sensors

This section presents recent advances in humidity sensors whose capacitance varies with relative humidity changes. As mentioned above, capacitance is considered a more complex physical quantity concerning reliable measurement—acquisition, with the required electronics design adding a considerable overhead in an end application when deploying such devices. Nevertheless, research results show that performance-wise, this type of sensors are notable and should always be considered. One of the first to discuss utilization of the substrate as active material was Harrey et al. [34] in 2002, where Ag electrodes were offset printed onto polyimide and polyethersulphone substrates and the substrates’ own capacitance was used for detecting relative humidity variations.

#### 2.2.1. Kapton—Based Capacitive Printed Humidity Sensors 

**Figure 10:**
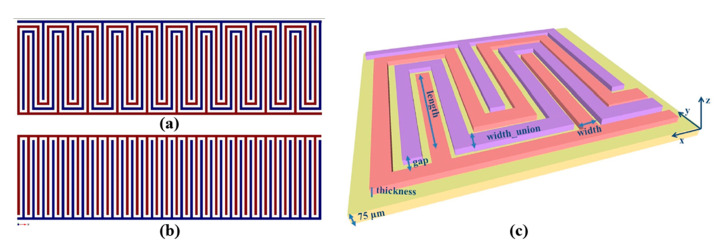
Serpentine (**a**) and IDE (**b**) geometries designed in the same area; design parameters for the novel proposed capacitor onto Kapton substrate (**c**) (reproduced with permission from [35] copyright 2014, Elsevier).

Rivadeneyra et al. [35] proposed a serpentine geometry (Figure 10) for an inkjet-printed capacitor electrode for humidity sensing and compared it against a typical interdigitated electrode array, finding a slightly better performance (1 fF/% RH better sensitivity, from 4.2 to 5.2 fF/% RH). This novel geometry improved capacitance by 21% for 180 fingers in the same given area by only printing 3% more material with respect to a typical IDE geometry.

**Figure 11:**
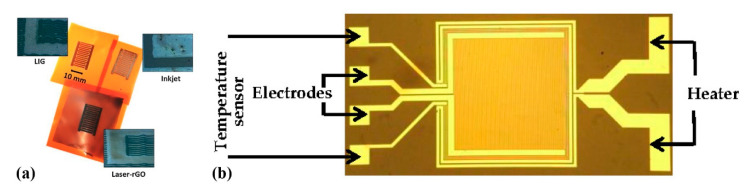
(**a**) The same geometry can be developed on Kapton by either inkjet printing, and laser-patterned rGO or graphene (reproduced with permission from [36] copyright 2019, Elsevier); (**b**) a set of electrodes developed via photolithography incorporating a heater and a temperature sensor (reproduced with permission from [37] copyright 2018, MDPI AG).

Romero et al. [36] recently compared development of a capacitive humidity sensor on Kapton via inkjet printing Ag lines for a fully printed sensor versus similar electrode geometries developed by laser-induced graphene and graphene oxide (Figure 11a). The authors conclude that the printed device exhibits the highest sensitivity and can operate at higher frequency. An alternative approach which incorporates polyimide has been also investigated [37]; a set of electrodes was developed with standard photolithography on a glass substrate and polyimide film was spin-coated on top (Figure 11b). This design also incorporates a heater for assisting in humidity desorption and a temperature sensor.

#### 2.2.2. PET—Based Capacitive Printed Humidity Sensors 

**Figure 12:**
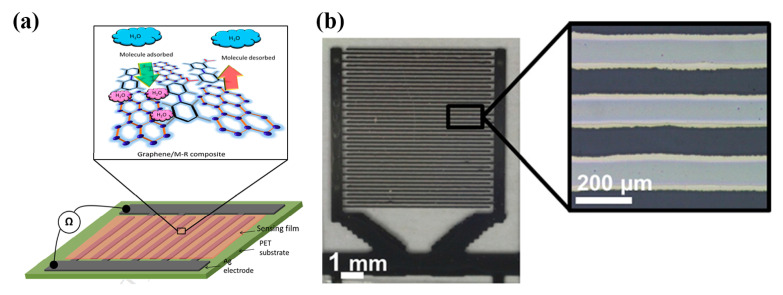
(**a**) Graphene/methyl-red composite sensor printed on PET substrate. The sensor exhibits both resistive and capacitive response to humidity variations (reproduced with permission from [20], copyright 2016, Elesevier); (**b**) An inkjet-printed capacitor coated with a protective Parylene-C film on PET (reproduced with permission from [39] copyright 2013, Elsevier).

The graphene/methyl-red composite-based sensor [20] has been also investigated for capacitive changes with humidity and has presented outstanding response: in the full range (5 to 95% RH), the sensor showed a capacitance change of 2869500% with a response time of 0.251 s (Figure 12a). Poly(methyl methacrylate) (PMMA) has been used as a humidity sensitive hydrophilic in PET-based devices [38]. Altenberend et al. [39] conducted a throughout investigation of the response of inkjet-printed Ag electrodes on PET substrates and their capacitive response for various relative humidity levels (Figure 12b). Furthermore, different strategies for sensing layer development and passivation have been examined, such as sintering conditions of Ag, oxidizing Ag with Ni, overlaying with Parylene-C and PEUT (poly(ether urethane)), resulting in enhanced long-term stability. Similarly, cellulose acetate butyrate and parylene have been utilized as sensing—passivation layer in paper-based humidity sensors [40]. McGhee et al. [41] presented a capacitive printed humidity sensor by using in-house indium tin oxide (ITO) and aluminum oxide (Al_2_O_3_) inks for screen printing. The sensors’ properties were tailored by using different design sizes. Another interesting development regarding custom ink development was the work recently published by Rivadeneyra et al. [42]: carbon dots (Cdots) were synthesized using dissolved citric acid and polyethyleneimine (PEI) in water, followed by drop-casting of the active layer on screen-printed Ag electrodes.

#### 2.2.3. Capacitive Humidity Sensors Comparison

An overview of the devices based on capacitive output along with their main attributes are presented in Table 3 for a comparative view of the state of the art. 

**Figure 13:**
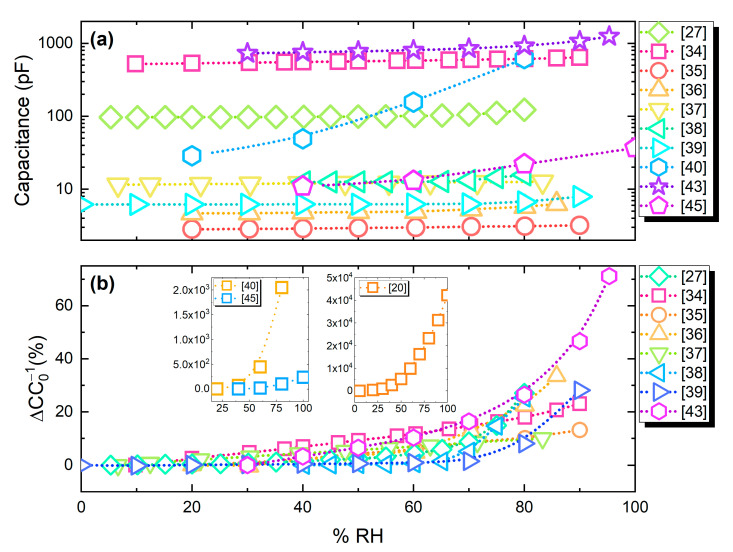
(**a**) Absolute response of capacitive humidity sensors; (**b**) relative capacitance change (%).

Figure 13a presents capacitance output values of the analyzed publications. The majority of the devices exhibits a comparable capacitance range; nevertheless, as seen in Figure 13b, a number of works report much higher percentage change under humidity variations.

## 3. Printed Temperature Sensors

**Figure 14:**
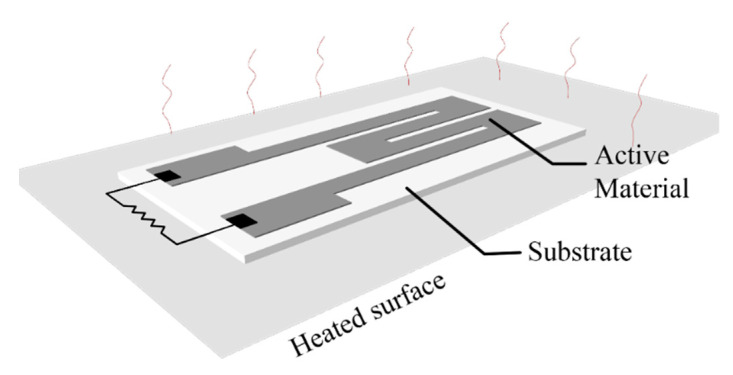
A typical setup for a printed temperature sensor evaluation.

A majority of printed thermometers consist of printed elements whose resistance—temperature relationship is either positive (resistance temperature detectors—RTD metallic inks and positive thermal coefficient—PTC materials) or negative (negative temperature coefficient—NTC materials). The main difference between RTD and PTC materials is that the later have faster response to temperature variations, but they exhibit a smaller sensing range. A lot of recently published research on printed temperature sensors focuses on e-skin—human temperature monitoring [46,47,48,49,50,51,52,53,54]; mainly because flexible substrates and inks are not tolerant to exposure to constant high temperatures (keeping in mind that inks are treated in specific temperatures for sintering), flexible substrates are an appropriate mean for mounting sensors onto human skin and organic materials used are fully biocompatible. A general scheme for a printed temperature sensor is presented in Figure 14: a printed active material which has either positive or negative temperature coefficient of resistance (TCR) is deposited onto a substrate, and for leveraging the substrate flexibility, it is common that the temperature detection to be performed on the back-plane; this way, the sensor under development targets to monitor temperature variations in a surface that its mounted onto (possibly non-planar). Dominant geometry in published works is a classic meander with varying width and total length.

### 3.1. Printed Temperature Sensors Based on PEDOT:PSS

**Figure 15:**
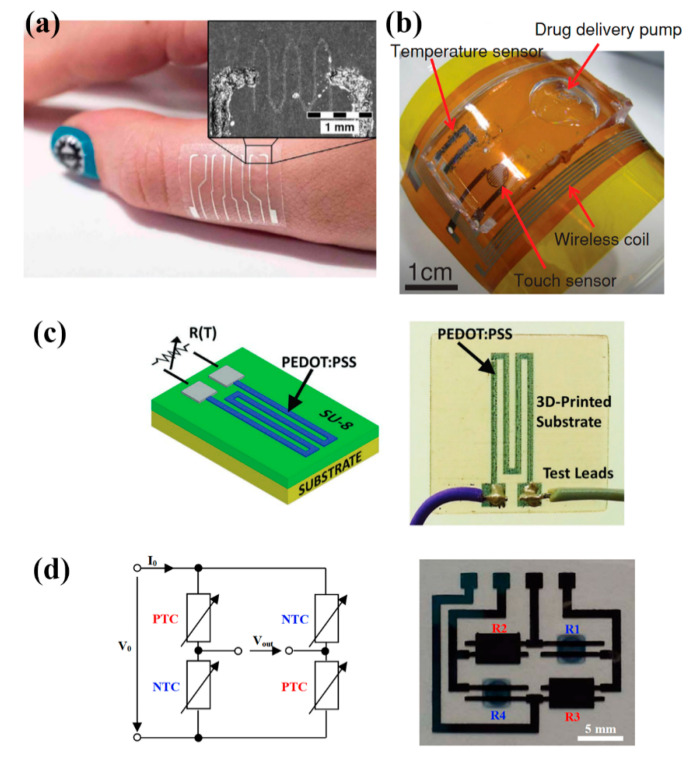
Various PEDOT:PSS-based temperature sensors, demonstrating a wide range of applications: (**a**) on-body temperature measurement patches (reproduced with permission from [46] copyright 2016, Springer-Nature); (**b**) (reproduced with permission from [47] copyright 2014, Wiley); (**c**) different substrates such as 3D-printed structures can be utilized as well (reproduced with permission from [56] copyright 2017, Wiley); (**d**) printed thermistors on PEN in bridge configuration (reproduced with permission from [55] copyright 2016, Elsevier).

Poly(3,4-ethylenedioxythiophene) polystyrene sulfonate (PEDOT:PSS) [55,56] PEDOT:PSS—carbon nanotubes (CNT) solutions [47,48,49,50,51,54] and graphene/PEDOT:PSS [46] solutions are printable, temperature sensitive organic materials and are commonly utilized for developing temperature sensors. Vuorinen et al. [46] presented inkjet—printed NTC thermistors composed of graphene and PEDOT:PSS and the sensor’s electrical contacts were composed by screen printing silver ink, all on adhesive bandage substrate. The sensor was evaluated in the range of 35 to 45 °C and TCR was measured to be an average of 0.047%/°C (Figure 15a). Honda et al. [47] reported a printed integrated system, a “smart bandage” on Kapton substrate, which incorporated PEDOT:PSS/CNT paste for measuring temperatures between 22 and 50 °C with a sensitivity of 0.61%/°C. (Figure 15b). A multifunctional system which includes a screen-printed PEDOT:PSS/CNT temperature sensor with a sensitivity of 0.89%/°C is presented in [48] as well. Yamamoto et al. [52] screen-printed PEDOT:PSS/CNT on pre-patterned PET substrate for a human body temperature sensor as well; this system also incorporates an accelerometer and ECG electrodes. PEDOT:PSS has been demonstrated to be printable onto other substrates such as a 3D printed structure for environmental parameters monitoring [56] (Figure 15c). Bali et al. [55] evaluated both a mixture of PEDOT: PSS and dimethyl sulfoxide (DMSO) (0.3–40 wt%), and a carbon nanoparticle ink, as inkjet—printed NTC and PTC temperature sensors on PEN respectively with findings indicating that both approaches offer good performance on temperature sensing (Figure 15d).

### 3.2. Printed Temperature Sensors Based on Ag

**Figure 16:**
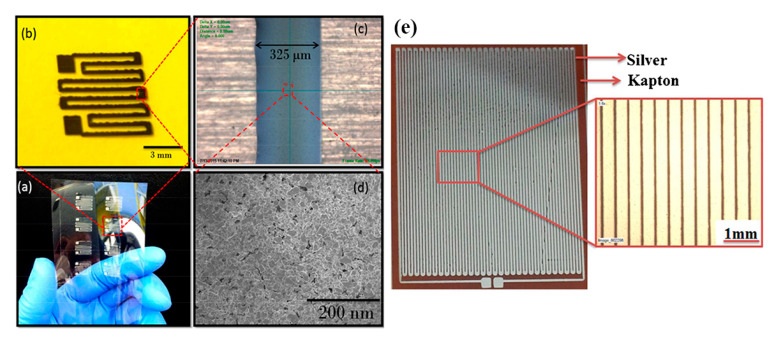
Ag-based temperature sensors: (**a**–**d**) a back-to-back topology for compensation of bending effects on temperature sensing (reproduced with permission from [59] copyright 2016, American Chemical Society); (**e**) Kapton-based inkjet-printed temperature sensor (reproduced with permission from [58] copyright 2016, Elsevier).

Silver-based temperature sensors are popular, given the fact that Ag is easily printed in various forms and is compatible with a variety of substrates. Bulk Ag temperature coefficient of resistance is 3.819 × 10^–3^ °C^−1^; it is each design-based approach and geometry that enhances the sensor performance alongside with possible combination with other materials. Courbat et al. [40] inkjet-printed lines of Ag NP-based ink on paper for the realization of both humidity and temperature sensors with a TCR of 0.0011 °C^−1^; also, it has been exhibited that overcoating with parylene did not influence temperature sensing properties. Similarly, Zikulnig et al. [57] printed and evaluated an Ag meander geometry on different types of paper; Kapton has also been proven to be an effective substrate for Ag-based temperature sensors; Dankoco et al. [58] inkjet-printed an organic silver complex compound ink. The corresponding results include a TCR of 2.19 × 10^–3^ °C^−1^ in the range of 20–60 °C (Figure 16e). Different measurement modes are discussed in recent publications [59,60], for example temperature measurements with differential temperature sensors integrated back–to–back for comprehension of bending effects [59] (Figure 16a–d).

### 3.3. Printed Temperature Sensors Comparison

Table 4 presents the most important parameters for materials, fabrication and sensing of various printed sensors.

**Figure 17:**
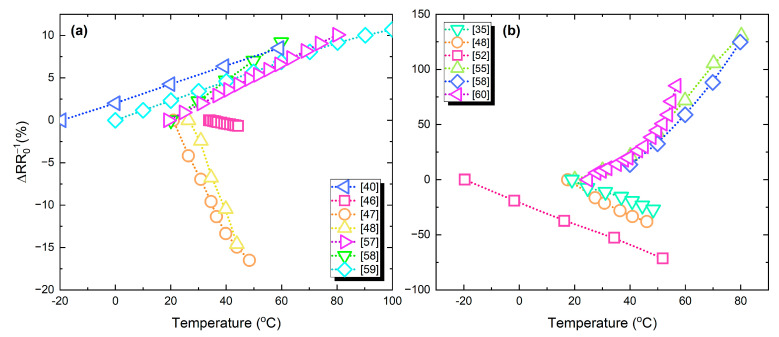
Temperature sensors relative resistance change to temperature variations: (**a**) devices with relatively lower response; (**b**) higher response.

Figure 17a,b present the percentage resistance change versus temperature; in Figure 17a devices with relatively lower response are grouped, while Figure 17b includes devices that exhibit a relatively higher response. Ag-based temperature sensors present a lower response, due to the metallic nature of the material which has a relatively low TCR.

## 4. Printed Strain—Bending Sensors

**Figure 18:**
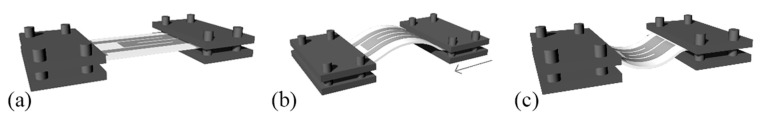
Different modes of mechanical deformation of a sample at rest (**a**); (**b**) tensile stress and (**c**) compressive stress.

Flexible strain—bending sensors are a blooming sensor category, mostly because of the exponential growth of smartphone and wearable electronics market. Common application of such type of sensors are in medical (e.g., minimally invasive surgeries) and automotive fields, in physical activity measurements and in human–machine interactions as well as in robotics. In this section, the literature review includes pressure, tactile and bending sensors, given the fact that depending on the installation and the application, the same sensor can be utilized both for sensing pressure and stress up to a degree of deformation. Mechanical stress discussed herein can either be compressive or tensile (Figure 18). Shear stress and other modes of deformation are not analyzed in this review.

Recent developments in additive manufacturing techniques and materials allow for realization of different complex structures for mechanical deformation sensing; there are various modes of operation, depending on the exploited physical phenomenon. There are three generally utilized groups based on either capacitive, piezoresistive or piezoelectric principle of operation. For capacitive sensing of mechanical deformation, a patterned capacitor exhibits alternation in its capacitance under deformation, due to distance changes between its electrodes (or plates); it is known that capacitance depends on the area of the plates, the distance between the plates and the dielectric constant of the material between the plates. On the other hand, piezoelectric and piezoresistive materials form the basis of the sensing layer by producing an electric field proportional to deformation for piezoelectric materials and a change in nominal resistance proportional to a deformation for piezoresistive materials. In a plethora of published works in strain and bending sensor manufacturing a printing process has been involved, for either partial or fully printed devices.

### 4.1. Capacitive Printed Strain—Bending Sensors

**Figure 19:**
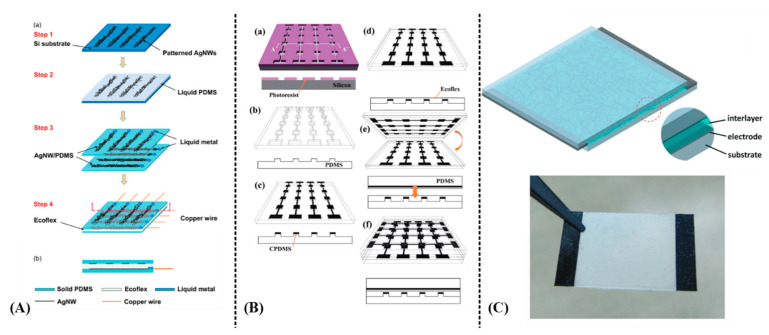
Various capacitive-based strain and pressure sensors: (**A**) a PDMS/AgNW/Ecoflex structure: device fabrication process (**a**); cross-sectional view of the device (**b**) (reproduced with permission from [69] copyright 2014, The Royal Society of Chemistry); (**B**) a PDMS/CNT/Ecoflex structure utilizing μcontact printing for fabrication. Micro-mold formation (**a**); PDMS stamp replication (**b**); CPDMS pattering via micro-contact printing (**c**); Ecoflex spin-coating (**d**); device assembly (**e**); thermal curing (**f**) (reproduced with permission from [70] copyright 2014, The Royal Society of Chemistry); (**C**) a different approach utilizing AgNWs and polyurethane on flexible substrate (reproduced with permission from [71] copyright 2015, The Royal Society of Chemistry). Note: the lowercase letters in the figure are kept from the original works, therefore the authors herein used capital letters for indication of each corresponding subfigure.

Yao and Zhu [69] reported capacitively sensing pressures of up to 1.2 MPa and 50% strains induced by tensile bending with AgNW/PDMS films and Ecoflex as dielectric material. Although response time was fast (~40 ms), a drawback in the reported sensor’s response was that sensitivity changes halfway through the measurements (1.62 MPa^−1^ between 0–0.45 MPa and 0.57 MPa^−1^ from 0.45 to 1.2 MPa for pressure sensing) (Figure 19A). Similarly, Woo et al. [70] presented a CNT/PDMS microprinted onto Ecoflex geometry in order to sense both tensile bending and pressure (Figure 19B). AgNWs have been also incorporated in a different geometry, forming a capacitor with polyurethane onto PET and flexible bandage substrates; this ink was spin coated but its rheological properties allow for direct replacement of spin coating with a printing process [71] (Figure 19C). Capacitive strain—bending sensors in a matrix topology are an excellent candidate for touch sensing and multi-button array for user input—tactile input for electronic devices.

### 4.2. Piezoelectric Printed Strain—Bending Sensors

**Figure 20:**
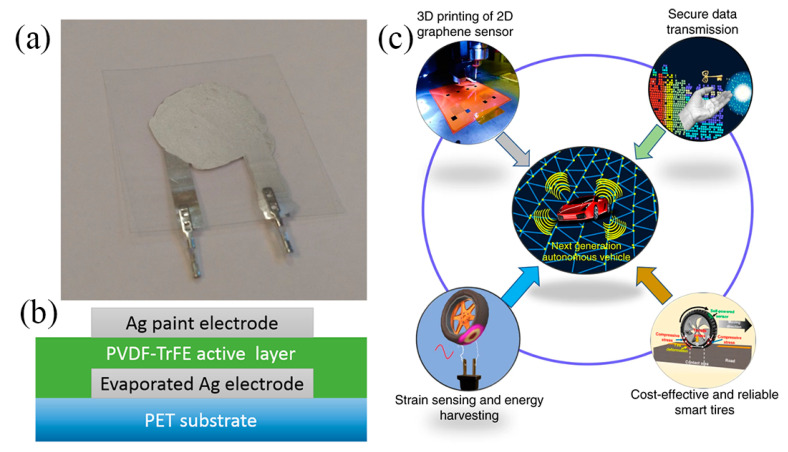
(**a**) A P(VDF-TrFE)-Ag sensor on PET substrate and (**b**) its cross-section (reproduced with permission from [72] copyright 2015, American Chemical Society); (**c**) a printed rGO strain sensor powered by PVDF integrated on a standalone tire monitoring system (reproduced with permission from [75], copyright 2020, Springer-Nature).

Piezoelectric output sensors are based on a piezoelectric material, the voltage output of which is proportional to mechanical deformation. Voltage output is regarded as susceptible to electrical noise under circumstances; therefore this category of sensors requires electronics for interfacing and signal conditioning. Also, polarization of the devices is required for operation [72]. Nevertheless, piezoelectric materials such as Polyvinylidenefluoride (PVDF) and poly(vinylidenefluoride-co-trifluoro-ethylene) [P(VDF-TrFE)] have been successfully utilized for developing such sensors and their output scale is adequate (tens of mV). A PVDF foil sensor with inkjet-printed Ag electrodes is presented in [73]; a problem which negatively influenced sensitivity was the sintering temperature (150 °C) of the printed electrodes which was close to the melting point of PVDF (175 °C), resulting in sensing layer shrinkage by approximately 15%. P(VDF-TrFE) has been successfully printed on PET substrate and alongside with a bottom Ag vacuum-evaporated electrode formed a highly sensitive sensor on bending mode [72] (Figure 20a,b). The fabrication steps can be replicated with other printing techniques towards a fully printed approach. For example, Khan et al. [74] fabricated sensors with similar material combination (P(VDF-TrFE)—Ag electrodes) on PET. This topology exhibits adequate sensitivity to produce a multi-touch array of sensors on flexible substrate, all using solely screen-printing technology. Recently, PVDF piezoelectric properties have been exploited to power a self-powered strain rGO printed sensor, integrated into an actual tire for real-time tire pressure monitoring [75]. (Figure 20c).

### 4.3. Piezoresistive Printed Strain—Bending Sensors

**Figure 21:**
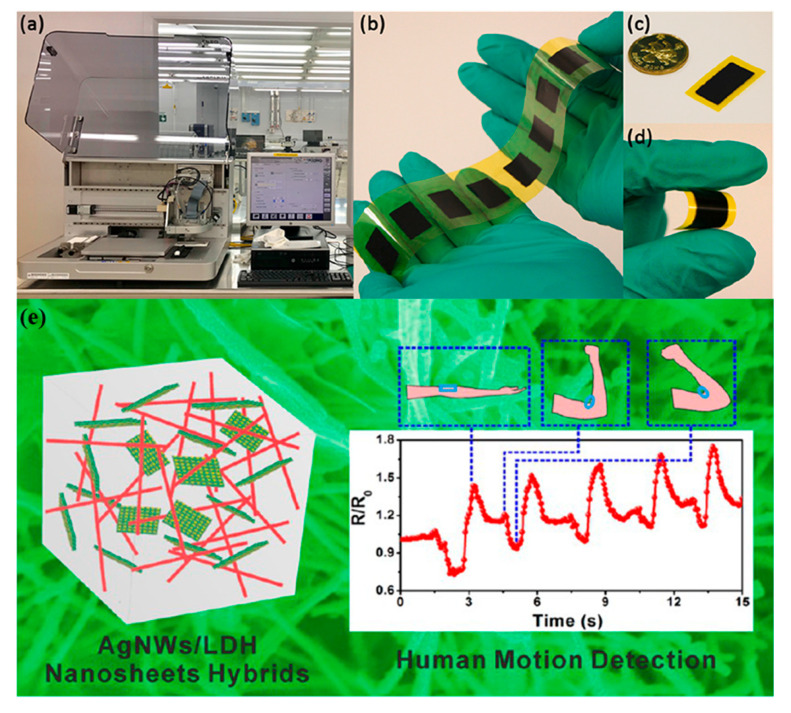
The printer (**a**) and (**b**–**d**) an inkjet-printed on Kapton piezoresistive dynamic strain sensor based on carbon black and polyvinyl pyrrolidone; (reproduced with permission from [77], copyright 2019, Elsevier); (**e**) an AgNW/LDH composite sensor for detection of different movements on human arm (reproduced with permission from [78] copyright 2015, American Chemical Society).

Piezoresistive sensors are the simplest in terms of readout and accompanying electronics needed; solely resistance measurements are adequate for extracting information regarding the mechanical deformation applied to the sensor. Khan et al. [74], alongside with a piezoelectric sensor presented an Ag/PDMS/MWCNT sandwich structure of piezoresistive sensors. The principle of operation is based on two factors: (a) the nanoscale changes induced by deformation in the MWCNTS and (b) the modulation of conductive paths in the matrix. It was found that lesser MWCNT concentration led to higher sensor response, alongside with a linear full-scale behavior. Xiao et al. [76] recently presented a screen-printed sensor on PI substrate based on polyvinyl chloride/carbon black composite with silver electrodes for sensing both tensile and compressive bending with a maximum angle of 100° corresponding to a relative length change (or strain) of 0.14%. The authors prior to bending experiments applied a repetitive stress to the sensor film in order to create microcracks which greatly enhanced performance, because contact resistance between cracks changes depending on the crack gap, which in turn changes with different strain. A high-frequency dynamic strain sensor was recently developed using inkjet printing with a composite ink consisting of carbon black nanoparticles and polyvinyl pyrrolidone; the sensor was capable of detecting dynamic strains up to a frequency of 500 kHz [77] (Figure 21a–d). Wei et al. [78] formed AgNW-layered double hydroxide (LDH) hybrid composites, which they patterned on paper substrate. The results show durability over 3000 bending cycles of 180°, fast response time (120 ms) and good sensitivity (0.16 rad^−1^) (Figure 21e). This system, owing to its nontoxicity and low-cost is a great demonstrator for mass producible, bending sensor. A multi-layer array design consisting of four different layers, two conductive, one insulating and one sensing [79]. This study presents important technical details for a fully printed device, where it is needed to selectively cover areas of conductive tracks, thus creating equivalent to traditional design vias.

### 4.4. Comparison of Printed Strain—Bending Sensors

Table 5 includes various bending/strain and pressure sensors alongside their key factors for manufacturing and sensing. Compared to the other categories, this type of sensors requires a more complex combination of both materials and processes. Also, design considerations are more demanding in terms of accurately laying out the appropriate structures for measuring strain and/or pressure.

## 5. Conclusions—Future Outlook

This review paper aimed at gathering, filtering, categorizing and presenting information regarding contemporary applications of printing technologies for sensor development. More specifically three types of sensors have been analyzed, namely humidity, temperature and strain sensors. For each sensor category, a literature review has been presented, alongside with representative examples for the development strategy, material selection and performance of the devices. 

Humidity sensors nominally share a common strategy: electrode deposition with a printing technique such as inkjet [12,17,20,35,36,39,40,45], screen [10,13,15,16,21,24,25,32,43] or gravure [14,38,44], or a traditional process such as lithography [26,27,28,37], followed by a sensing layer deposition either via inkjet [22,23,40], screen [15,18,25,32,43], gravure [10,13,16,21,44], spin coating [27,28,37,38] or drop casting [14,19,26]. Some groups have presented an approach where the substrate acts as a sensing layer [12,17,34,35,36,45,87] thus eliminating the need for an additional step of deposition of such layer, while humidity sensors can either provide a resistive or capacitive output (Table 1 and Table 2, respectively). Temperature sensors usually exploit a specific material’s thermal properties, and more specifically thermal coefficient of resistance (TCR), thus requiring only one material for sensor development. Various works utilize inkjet or screen-printed silver [17,40,57,58,59,60], PEDOT: PSS (pristine or in combination with graphene or CNTS) [17,46,47,48,52,55,56] amongst other materials which exhibit either positive or negative TCR. Mechanical deformation sensors analyzed in this review have the largest diversity both in terms of material selection and technologies required for manufacturing. This type of sensors can either provide a capacitive, piezoelectric or piezoresistive output. In most cases, an elastomer such as PDMS [69,70,74,82,83,84,85] in conjunction with a conductive material such as MWCNT form a matrix which can be either directly utilized as a sensor, or with the additional printing of conductive electrodes onto it [69,71,72,74,76,79,80,84,85]. PVDF—P (VDF-TrFE) has also been utilized as a piezoelectric element in piezoelectric output sensors [72,73,74,80].

Printing technologies such as inkjet printing and screen printing, are evidently being adopted by numerous scientific groups for partial or full development of electronic devices development, owning a set of characteristics such as large-scale and low-cost fabrication, variety of compatible materials and substrates and rapid prototyping implementation. By leveraging the aforementioned advantages, printed electronics for humidity, temperature and strain sensor development can act as a platform for future turnkey production, all by satisfying requirements such as substrate flexibility, multi-material deposition detection.

Application-wise, most of the works presented herein fall in the scope of either environmental monitoring or wearable-biomedical metrics from on-body devices. Nevertheless, it was observed that evaluation was mainly performed using laboratory measurement equipment. It is therefore concluded that a wide field for future development is the interfacing electronic circuit for data measurements and transmission to an application front-end, towards realization of a printed-based system on flexible substrate, which can implement either printing or traditional patterning techniques. Challenges to be addressed concerning integration of such devices are (a) the soldering and interconnections of discrete electronic components such as SMT resistors and microcontroller on the same substrate with the printed sensors [88], (b) passivation of the devices for long-term endurance in field applications outside the laboratory and (c) development of dedicated, low-power, high-performance electronics for signal acquisition and conditioning. By effectively tackling these challenges, a revolutionary era for the development of printed sensors for consumer applications will be closer to realization.

## Figures and Tables

**Table 1:** Search results (publications) from Web of Science for “Printed Humidity Sensor”, “Printed Temperature Sensor” and “Printed Strain Sensor” between 2013–2020.

Query	Results Found	Sum of the Times Cited	Avg. Citations Per Item	H-Index
Printed Humidity Sensor	381	5055	13.27	37
Printed Temperature Sensor	1513	21,968	14.52	60
Printed Strain Sensor	774	17,970	23.22	62

**Table 2:** Comparison οf resistive humidity sensors.

Active Material	Electrodes	Substrate	Range (%RH)	Sensitivity ^1^	Ref.
Gravure printed MWCNTs	Screen-printed, Ag	PI	30–60	0.96%/% RH	[10]
Gravure printed MWCNTs	Screen-printed, Ag	PI	10–90	–	[13]
Drop-cast TiO_2_ nanoflowers	Gravure, Ag	PI	20–95	485.7/RH%	[14]
Screen-printed MEPAB/CMDAB/MMA copolymer	Screen-printed, Ag/Au	PI	20–95	0.0586 logΩ/% RH	[15]
Screen-printed epoxy/IPN polyelectrolyte	Chemical Etching–Plating (Ni/Au)	PI	20–95	0.046 logΩ/% RH	[18]
Drop-cast SnO_2_/rGO	Chemical Etching–Plating (Cu/Ni)	PI	11–97	15.19–45.02%	[19]
Spin-coat PEDOT:PSS (15%) + PVA (SAW)	Photolithography	LiNbO_3_	0–80	350 Ω/% RH	[27]
Spin-coat PEDOT:PSS (5 wt%) + ZnSnO_3_ (5 wt%)	Photolithography, Au	LiNbO_3_	0–90	–	[28]
Screen-printed MDBBAC/MMA (70/30) Polyelectrolyte	Screen-printed, Ag–Plating (Cu/Ni/Au)	Glass Epoxy	20–95	0.0349 logΩ/% RH	[25]
EHD Graphene/methyl-red	Inkjet-printed, Ag	PET	5–95	96.36%	[20]
Drop-cast Pt/MoS_2_ (0.25:1)	Photolithography, Au	Ceramic	35–85	~4000 times (85 % RH)	[26]
Gravure printed CNT	Screen-printed, Ag	PET	20–80	0.1%/% RH	[16]
Screen-printed TiO_2_-Cu_2_O-Na_2_O	Screen-printed, Pt	Al_2_O_3_	20–95	–	[32]
Inkjet-printed PANI	–	Polyester	20–95	–	[23]
Micro-pipette deposited Nafion	Screen-printed Ag on screen-printed PU	Polyester Cotton Fabric	30–90	–	[24]
Gravure printed FMWCNT/HEC (1:6 *w*/*w*)	Screen-printed, Ag	PET	20–80	0.048/% RH	[21]
Inkjet-printed PEDOT:rGO-PEI/Au NPs	–	PET	11–98	7.41–51.60%	[22]
Spin-coated Fe_2_O_3_	Inkjet-printed, Ag	PET	0–100	~88.89%	[33]
Substrate	Inkjet-printed, Ag	Paper	18–88	–	[12]
Substrate	Inkjet-printed Ag & PEDOT: PSS	Paper	0–85	0.0492 & 0.0551 logΩ/% RH	[17]

^1^ as reported. Some reports provide only a percentage which corresponds to total change for the full range, e.g., total change in resistance from 20 to 80% RH (min—max change).

**Table 3:** Capacitive Humidity Sensors.

Active Material	Electrodes	Substrate	Range	Sensitivity ^1^	Reference
7.6 μm polyimide (HN30) (Substrate)	Offset, Ag	PI	10–90	1.025 pF/% RH	[34]
75 μm polyimide (Substrate)	Inkjet, Ag	PI	20–90	5.2 ± 0.2 fF/% RH	[35]
125 μm polyimide (Substrate)	Inkjet, Ag	PI	20–85	24.71 fF/% RH	[36]
4.6 μm spin-coated polyimide	Lithography, Au	Glass	6–85	15.2 fF/% RH	[37]
PEDOT:PSS (5%) + PVA (SAW)	Photolithography	LiNbO_3_	0–80	0.33 pF/% RH	[27]
Spin-coated PMMA	Gravure, Ag	PET	40–80	11.9%	[38]
EHD Graphene/methyl-red	Inkjet, Ag	PET	5–95	2869500%	[20]
Ni/Parylene-C/Poly(etherurethane)	Inkjet, Ag	PET	10–90	3.15 fF/% RH	[39]
Inkjet, cellulose acetate butyrate	Inkjet, Ag	Paper	20–80	–	[40]
Screen-printed PDMS-CaCl_2_	Screen-printed Ag/Au	Textile	30–95	10.2% (30 to 60% RH)	[43]
Gravure, pHEMA	Gravure, Ag	PET	30–80	172%	[44]
Screen-printed Indium Tin Oxide/Aluminum Oxide	Screen-printed Ag	PET	5–95	0.85–7.76 pF/% RH	[41]
Drop-cast, Carbon dots	Screen-printed Ag	PET	20–90	70 fF/% RH	[42]
Substrate	Inkjet, Ag	Paper	40–100	2 pF/% RH	[45]

^1^ as reported. Some reports do not provide the sensitivity but a percentage which corresponds to total change for the full input signal range, e.g., total change in resistance from 20 to 80% RH (min—max change).

**Table 4:** Printed temperature sensors.

Materials	Substrate	Process	Range (°C)	Sensitivity (%/°C)	Ref.
PEDOT: PSS/graphene/EGC	PU	Inkjet	35–45	0.064/0.034	[46]
PEDOT: PSS/CNT	PI	Shadow Mask	22–50	0.61	[47]
PEDOT: PSS/DMSO	PEN	Inkjet	20–70	2.5 × 10^−3^	[55]
PEDOT: PSS/CNT (3:1)	PET	Screen printing	26–45	0.89	[48]
PEDOT: PSS	SU-8	Inkjet	−20–50	0.018	[56]
PEDOT: PSS/CNT	PET	Screen printing	20–45	1.3	[52]
Ag	PI	Inkjet	20–60	2.19 × 10^−3^	[58]
Ag	PET	Inkjet	0–100	1.076 × 10^−3^	[59]
Ag	PET	Inkjet	30–100	0.1086 Ω/°C	[61]
Au, PTC & NTC pastes	PEN/PET	Screen printing	20–80	0.06 V/°C	[62]
Ag, Ni	PET	Inkjet, Electrodeposition	−10–60	1.82 × 10^−3^	[60]
Flake graphite/CNT/PDMS	PET	Screen printing	40–80	0.028	[63]
Mn_2_O_3_/NiO/Co_3_O_4_/CuO/ZnOPVDF, PDMS, CYTOP	PI	Screen printing	40–140	91.76% (full range change)	[64]
BaTiO_3_, activated carbon, thermoset polymeric	PET	Screen printing	25–55	0.022	[65]
MoSe_2_, Ag	Glass	Drop-cast	−0.15–99.8	~−0.51	[66]
Polylactic Acid—Carbon black	Free standing	3D Printing	25–36	–	[67]
Polyvinyl chloride/carbon black	PET	Screen printing	18–44	−0.148	[68]
Ag	Paper	Inkjet	−20–60	1.1 × 10^−3^	[40]
Ag	Paper	Inkjet	20–80	1.63 × 10^−3^	[57]
Ag & PEDOT: PSS	Paper	Inkjet	25–45	0.938 × 10^−3^ & −13.9 × 10^−3^	[17]

**Table 5:** Printed Mechanical Deformation Sensors.

Materials	Substrate	Process	Principle	Range	Sensitivity/Gauge Factor	Ref.
AgNWs, Ecoflex, PDMS	Embedded	Screen printing, drop-casting	Capacitive	50% tensile	GF: 0.7	[69]
CNTs, Ecoflex, PDMS	Embedded	μcontact printing	Capacitive	50% tensile	GF: 0.5	[70]
AgNW-PU	PET, Adhesive Bandage	Drop-cast, spin-coating	Capacitive	2 mm tensile	5.54 kPa^−1^–0.88 kPa^−1^	[71]
PVDF, AgNPs	Embedded	Inkjet	Piezoelectric	3 N	2.8 ± 0.9 mV/N	[73]
P(VDF-TrFE), Ag	PET	Vacuum evaporation	Piezoelectric	–	140 ± 50 nC/N	[72]
Ceramic dielectric, Pt/Ag	Steel	Screen printing	Piezoelectric	0–18 MPa	–	[80]
P(VDF-TrFE), Ag	PI	Screen printing	Piezoelectric	0.5–4N	0.05 V/N	[74]
Ionic liquid/polymer, MWCNT	TangoPlus	3D printing, molding	Piezoresistive	–	–	[81]
MWCNT, PDMS, Ag	Embedded	Screen printing	Piezoresistive	0–11 N Compressive	20 kΩ/N	[74]
Polyvinyl chloride/carbon black, Ag	PI	Screen printing	Piezoresistive	0.14%	GF_tensile_: 741 GF_compr_: 1563	[76]
Thin graphite nanobelt, Ti/Au	PDMS	Modified Langmuir-Blodge	Piezoresistive	40% Strain	GF: 1–24	[82]
PDMS, MWCNT	Embedded	3D printing	Piezoresistive	15% Strain	GF: 1–16	[83]
PDMS, MWCNT, Ag	PET	Screen printing	Piezoresistive	1.5 kPa–15.5 kPa	–	[84]
PEDOT: PSS/PUD, Ag	PDMS	Mold cast, inkjet	Piezoresistive	3 Pa–5 kPa	–	[85]
AgNW, Layered double hydroxides	Paper	Screen printing	Piezoresistive	180° Compressive	0.16 rad^−1^	[78]
PeDOT, TIPS-pentacene, Ag, PVPh	PI	Inkjet	Piezoresistive	4000 N	GF: 0.35	[79]
PEDOT: PSS & Ag	Paper	Inkjet	Piezoresistive	10%	GF_tensile_: 0.42 GF_compress_: 0.15	[17]
PEDOT: PSS	PET	Inkjet	Piezoresistive	0.33%	165	[86]

## Data Availability

The data presented in this study are available on request from the corresponding author.
